# Case of Compound Fractures of Both Legs, in Which a Bitter Infusion Was Used as a Lotion, with a View of Preventing the Formation of Maggots

**Published:** 1826-11

**Authors:** 

**Affiliations:** St. George's Hospital.


### FRACTURE OF THE LEGS.

*Case of Compound Fractures of both Legs, in which a Bitter Infusion was used as a Lotion, with a view of preventing the formation of Maggots.* Treated by Mr. Rose, at St. George's Hospital.

Henry Daniels, a lad of fourteen years of age, was brought to St. George's Hospital, on the 24th of last July, with compound fractures of both his legs. He had fallen from a high branch of a tree, by which he had fractured the tibia and fibula of his right leg, nearly in the middle of these bones, and produced a lacerated wound of the soft parts on the fore part of the limb, opposite the interosseal ligament. This wound was at least four inches in length; its edges were separated to some extent, and could not be approximated; and about two inches, or rather more, of the tendon of the tibialis anticus, were torn from amongst the fleshy fibres of that muscle, and lay exposed in the wound. He had fractured the tibia of his left leg, also in its middle, and the fibula of that leg nearer the head of the bone. There was in this, as in the other limb, a very extensive lacerated wound of the integuments, in the same situation, and nearly, though not quite, so large as that in the right. In both legs, the fasciæ covering the muscles, and parts of the muscular fibres, were wounded and torn; but it was not ascertained whether this had been produced by any protrusion of the fractured bones, or by parts of the tree in his fall. The bones did not protrude when the boy was brought to the hospital, and he was too much alarmed and agitated to give any satisfactory account of what had taken place. The appearance of the wounds might be accounted for upon either supposition.

At the time of his admission, the wounds were bleeding freely. A thin piece of lint was laid over each of them, and they were afterwards covered with compresses moistened with infusion of quassia. The patient was laid on his back, and the limbs were placed in junks, by which the fractured bones were kept in proper apposition. The quassia was used in the hopes of its preventing the flies (which, in the excessively sultry weather which then prevailed, abounded in the wards,) from depositing their larvæ on the bandages and compresses, which were necessarily soon soaked with the blood and serum oozing from the injured parts. It fully answered this purpose, and, by so doing, contributed to the favourable result of the case.

The inflammatory fever was moderate, and the bandages and dressings were kept constantly wet with the same infusion, and were not removed until the 14th of August, three weeks from the time they were first put on. There was then complete soft union of all the broken bones; the ulcers in both legs were clean and healthy, covered with large spongy granulations, such as might be expected from membrane or bone. Splints and common rollers were applied; and on the 6th of September he was dismissed, the fractures being firmly united, and the wounds very nearly healed.

From the copious oozing of blood and serum in the first instance, and the discharge of fetid pus which afterwards took place, it would not have been easy to prevent the formation of maggots, if the common cooling lotions had been used; and, though the patient's age was very favourable, so speedy a cure could not have been expected, if any circumstance had rendered it necessary to disturb the fractured bones at an earlier period.

---

